# The peripheral soft tissues should not be ignored in the finite element models of the human knee joint

**DOI:** 10.1007/s11517-017-1757-0

**Published:** 2017-12-07

**Authors:** Hamid Naghibi Beidokhti, Dennis Janssen, Sebastiaan van de Groes, Nico Verdonschot

**Affiliations:** 1 Orthopedic Research Laboratory, Radboud Institute for Health Sciences, Radboud University Medical Center, 6525 GA Nijmegen, The Netherlands; 2 Orthopaedic Department, Radboud University Medical Center, Nijmegen, The Netherlands; 3 Laboratory of Biomechanical Engineering, University of Twente, Enschede, The Netherlands

**Keywords:** Finite element method, Knee laxity, Knee peripheral tissues, Knee posterior capsule, Kinematics

## Abstract

In finite element models of the either implanted or intact human knee joint, soft tissue structures like tendons and ligaments are being incorporated, but usually skin, peripheral knee soft tissues, and the posterior capsule are ignored and assumed to be of minor influence on knee joint biomechanics. It is, however, unknown how these peripheral structures influence the biomechanical response of the knee. In this study, the aim was to assess the significance of the peripheral soft tissues and posterior capsule on the kinematics and laxities of human knee joint, based on experimental tests on three human cadaveric specimens. Despite the high inter-subject variability of the results, it was demonstrated that the target tissues have a considerable influence on posterior translational and internal and valgus rotational laxities of lax knees under flexion. Consequently, ignoring these tissues from computational models may alter the knee joint biomechanics.

## Introduction

**Fig. 1:**
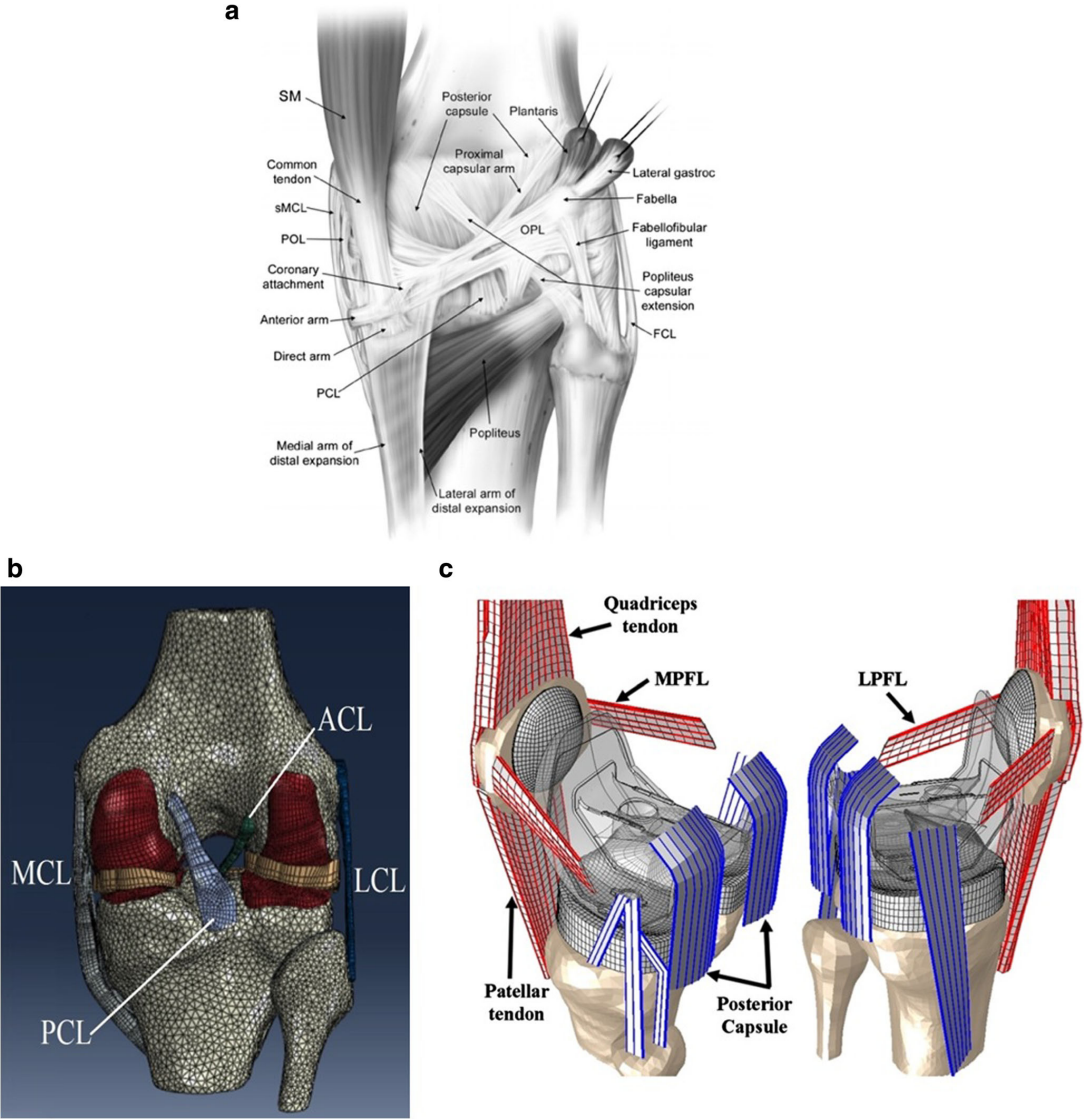
A posterior view of a schematic human knee joint (reproduced from [8] Elsevier license permission 3932521102554) (**a**); a typical FE model of a native knee joint (reused from [14], the original image was horizontally flipped and labeled) (**b**); and an FE model with posterior capsule inclusion (reproduced from [6] Elsevier license permission 3981261251500) (**c**)

The finite element (FE) method is being widely utilized as a research tool to investigate knee biomechanics [1]. However, every FE model of either native or implanted knees suffers from limitations and simplifications [2]. In even the most comprehensive FE model of the knee, soft tissue structures like tendons and ligaments are being incorporated, but usually skin, peripheral soft tissues, and the posterior capsule are ignored, mostly due to the lack of experimental data on their influence on the joint kinematics and laxity [3, 4] (Fig. 1). On the other hand, only a few studies modeled posterior capsule in either native (i.e., Shin et al. [5]) or implanted (i.e., Baldwin et al. [6]) knee models, roughly approximating the properties based on the limited experimental data of Brantigan and Voshell [7] (Fig. 1c). The influence of these peripheral structures on the biomechanical behavior of the knee joint is largely unknown and usually assumed to be of minor influence on the overall kinematics of the knee joint.

Geiger et al. reviewed the posterolateral and posteromedial soft tissue structures [8]. LaPrade et al. verified the quantitative anatomy of medial structures of the knee joint including the posterior oblique ligament [9]. None of them, however, assessed the properties of their target tissues. A few studies investigated the effect of the lateral soft tissues, and more importantly of the popliteofibular ligament and popliteal tendon, on varus and external rotational laxities under limited loading conditions [10–13]. Their results indicated that the popliteofibular ligament contributes to posterolateral stability [12] and prevents excessive posterior translation and varus angulation [11], especially when the knee is flexed [13]. Sugita et al. indicated that the popliteal tendon and popliteofibular ligaments are equally important in posterolateral stability of the knee [10]. Griffith et al. measured the oblique popliteal ligament (OPL) force at different loading conditions and indicated that it takes part in the internal and valgus rotational stiffness at low flexions [15]. Rachmat et al. estimated the mechanical properties of posterior capsule based on isolated ex situ uniaxial tensile tests [16]. Their results showed asymmetrical mechanical properties in the medial, central, and lateral regions. However, the outcome based on the isolated ex situ testing condition could only be correlated to a limited knee gesture (hyper-extension).

The influence of the peripheral structures and posterior capsule on knee joint laxity has not been completely described in the literature, but is of interest for computational modelers. The aim of this study, therefore, was to assess the significance of the peripheral soft tissues and posterior capsule on the kinematics and laxity of the human knee joint. Accordingly, a computational approach to model the target tissues in FE was sought.

## Materials and methods

### Experimental testing

**Fig. 2:**
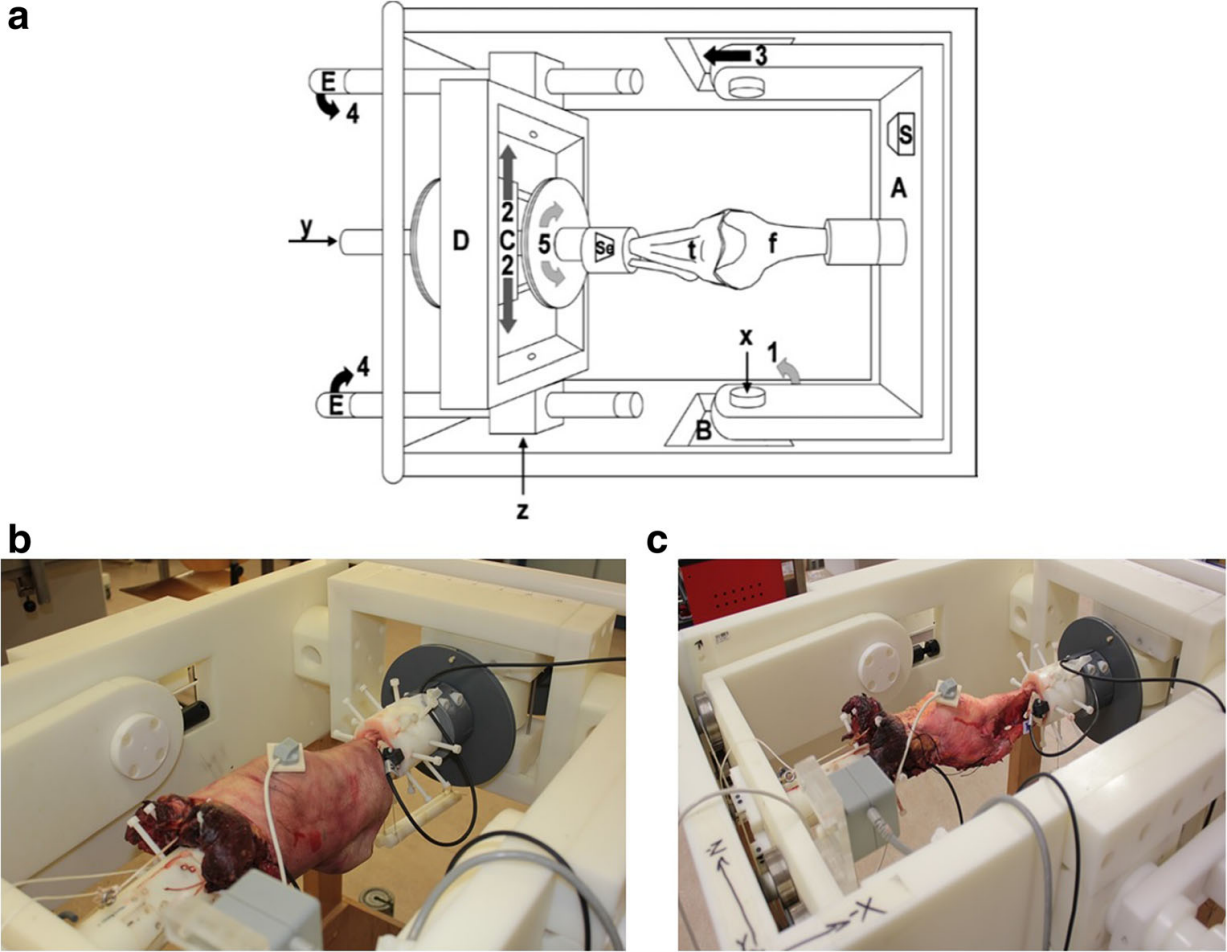
The six-DOF knee testing apparatus (**a**); a single knee joint positioned in the testing apparatus with the tracking sensors attached to bony segments: pre-dissection joint (**b**) and post-dissection joint (**c**)

Three fresh-frozen cadavers with a mean age of 79 ± 21 years, with no signs of hard and soft tissues injuries and no history of surgery were selected for the current study. The specimens were received from the Anatomy Department of Radboud University Medical Center with a permission statement for experimental use. The knees were prepared following a standard protocol and positioned in a knee testing apparatus that allows for six degree of freedom motions (Fig. 2a) [17–19]. Flexion-extension was applied to the femur, whereas the valgus-varus and internal-external rotations and anterior-posterior and medial-lateral translations were applied to the tibia.

The quadriceps muscles were subjected to constant forces provided by torsional springs representing the vastus lateralis (20 N), rectus femoris (20 N), and the grouped vastus medialis and intermedius (10 N) [17, 20, 21]. These loads were selected based on the force magnitude limitations of the knee testing apparatus and applied in order to stabilize the patella and, as a result, were not meant to be representative of quadriceps loads during in vivo tasks.

An electromagnetic tracking system (3Space Fastrak, Polhemus Incorporated, VT, USA) was used to track sensors that were rigidly attached to the femur, tibia, and patella, using base plates screwed onto the bone. Subsequently, the knees with the base plates in situ were CT scanned (Toshiba Aquilion ONE, Otawara, Japan) with a slice thickness of 0.5 mm and segmented using Mimics v18.0 (Materialise, Leuven, Belgium). The segmented three-dimensional models were used to determine the relative position and orientation of sensors with respect to the joint. In-house developed scripts (MATLAB R2013a, Natick, MA) were used to calculate the knee joint center (similar to [22]) and to convert the raw tracking data to kinematics in the knee joint coordinate system [23], as described by Grood and Suntay [24].

Six different loading conditions were applied to the intact knees (Fig. 2b) at four different flexion angles (0°, 30°, 60°, and 90°): internal and external torque of 5.16 Nm, a varus and valgus moment of 12 Nm, and an anterior and posterior load of 100 N. These loads were based on the literature values and can provide sufficient laxity motion to characterize the knee ligamentous structures without damaging the cadaveric specimens [6, 24–27]. The loads were applied within the physiological loading range (~ 1 s). The measurements were performed after ~ 3 s of external loading, and after which, the biomechanical response of the ligamentous structures of the knee joint would not considerably be influenced by the tissue viscoelasticity [28].

Subsequently, the knee joints were dissected by an experienced knee surgeon to remove the skin, peripheral soft tissues and posterior capsule, while preserving the salient tibiofemoral ligaments such as the anterior and posterior cruciate ligaments, and the medial and lateral collateral ligaments (Fig. 2c). Subsequently, the loading conditions as described above were repeated to determine the effect of the dissection of the peripheral soft tissue structures. Each of the loading conditions was repeated three times to check the repeatability of the measurements, and their mean and standard deviation were calculated.

### Finite element modeling

**Fig. 3:**
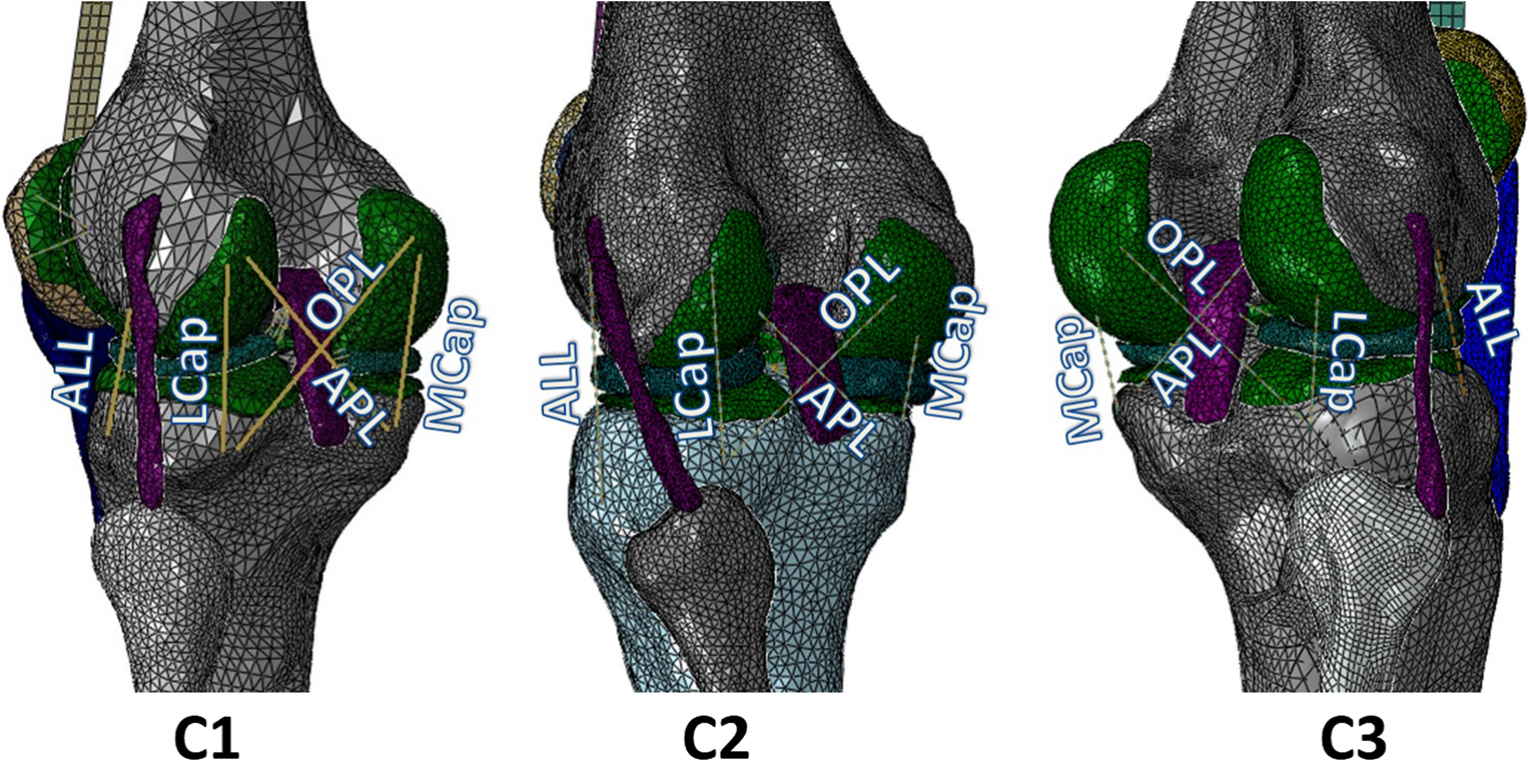
The validated subject-specific FE models of the three cadaveric knees (C1, C2, and C3) with five springs added to be representative for the dissected tissues as oblique popliteal ligament (OPL), arcuate popliteal ligament (APL), medial capsule (MCap), lateral capsule (LCap), and anterolateral ligament (ALL)

Three validated subject-specific FE models of the three dissected knees were developed in our earlier study [29]. Five structures were added to each FE model, including oblique popliteal ligament (OPL), arcuate popliteal ligament (APL), medial capsule (MCap), lateral capsule (LCap), and anterolateral ligament (ALL) (Fig. 3). The insertion sites were estimated from the segmented model and anatomy textbooks. All the structures were modeled as no-compression linear spring, and the initial stiffness was assigned from the literature [10, 11, 13, 29–32]. The stiffness of each structure was varied within the specified range to obtain the closest laxity prediction to the experimentally measured laxity in the intact knee, under the six loading regimes described previously. The same approach was previously used by Baldwin et al. [6].

## Results

In the following, the laxity outcomes of the specimens pre- and post-dissections were compared separately for anterior-posterior translational, internal-external rotational, and valgus-varus rotational laxities. Despite the large inter-subject variability in some directions, the average laxity changes following dissection (± standard deviations) of the three specimens have been included in Table 1. Subsequently, the peripheral soft tissue stiffness values were incorporated in the FE models. Finally, the FE laxity predictions with and without these additional structures were compared with the experimental measurement.

**Table 1:** Average laxity changes in the six loading conditions following the dissection (± standard deviations) for all three specimens

		Loading regimes					
		Internal torque (5.16 Nm)	External torque (5.16 Nm)	Varus moment (12 Nm)	Valgus moment (12 Nm)	Anterior load (100 N)	Posterior load (100 N)
		Internal rotation (°)	External rotation (°)	Varus rotation (°)	Valgus rotation (°)	Anterior translation (mm)	Posterior translation (mm)
Flexion angle (°)	0	0.5 ± 0.6	0.5 ± 0.2	0.3 ± 1.0	0.5 ± 0.8	0.0 ± 0.8	0.2 ± 1.3
	30	1.5 ± 0.8	0.2 ± 0.8	0.2 ± 1.0	0.9 ± 1.2	0.1 ± 0.5	0.1 ± 1.8
	60	6.0 ± 1.4	1.1 ± 1.7	0.0 ± 0.3	0.3 ± 1.2	0.3 ± 0.4	0.1 ± 0.8
	90	6.3 ± 3.5	0.9 ± 2.1	0.1 ± 0.6	0.3 ± 1.7	0.2 ± 0.6	0.4 ± 0.8

### Experimental laxities

#### Anterior-posterior laxity

**Fig. 4:**
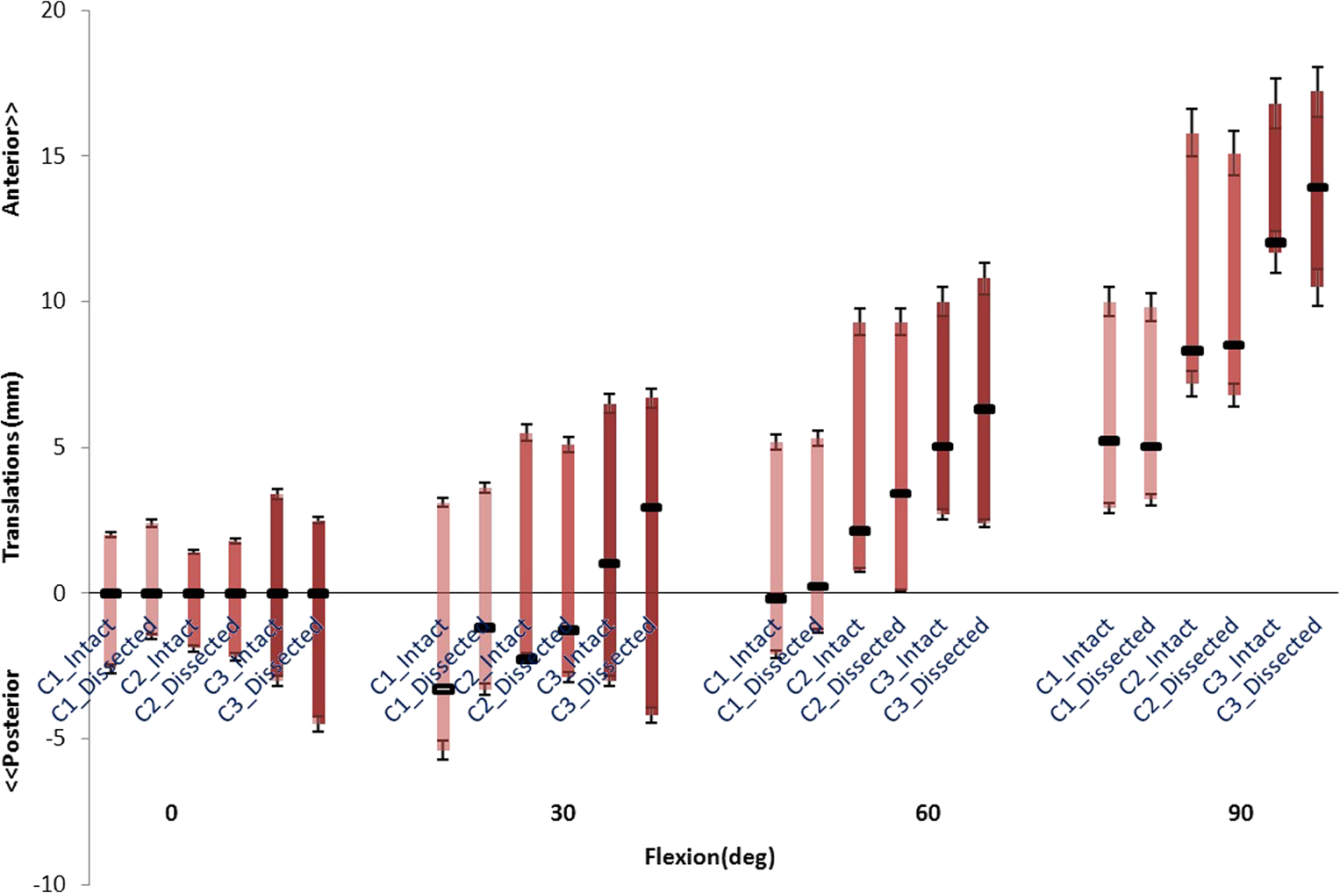
Anterior-posterior laxity of the three cadaveric knees at four flexion angles indicating unloaded (middle square), anteriorly loaded (upward bars), and posteriorly loaded (downward bars) cases

Figure 4 shows the anterior-posterior laxity in the three specimens, for the pre- and post-dissection cases.

All dissected knees showed a slightly larger average tibial anterior translation (1.0 to 2.1 mm at 30° and 0.4 to 1.3 mm at 60°). At 90°, the first two knees were negligibly affected by the dissection of the peripheral tissues, while the difference in the third knee was 1.9 mm. No considerable difference in anterior translation was found between the three knees.

Surprisingly, the posterior laxity of the first knee was reduced after dissection, although by less than 1.0 mm. In the second specimen, the posterior laxity increased by 1.7 and 1.0 mm at 30° and 60°, respectively. The third knee was more sensitive to peripheral soft tissues, as the posterior laxity at 0°, 30°, 60°, and 90° increased by 1.5, 3.1, 1.6, and 3.1 mm, respectively.

#### Internal-external laxity

**Fig. 5:**
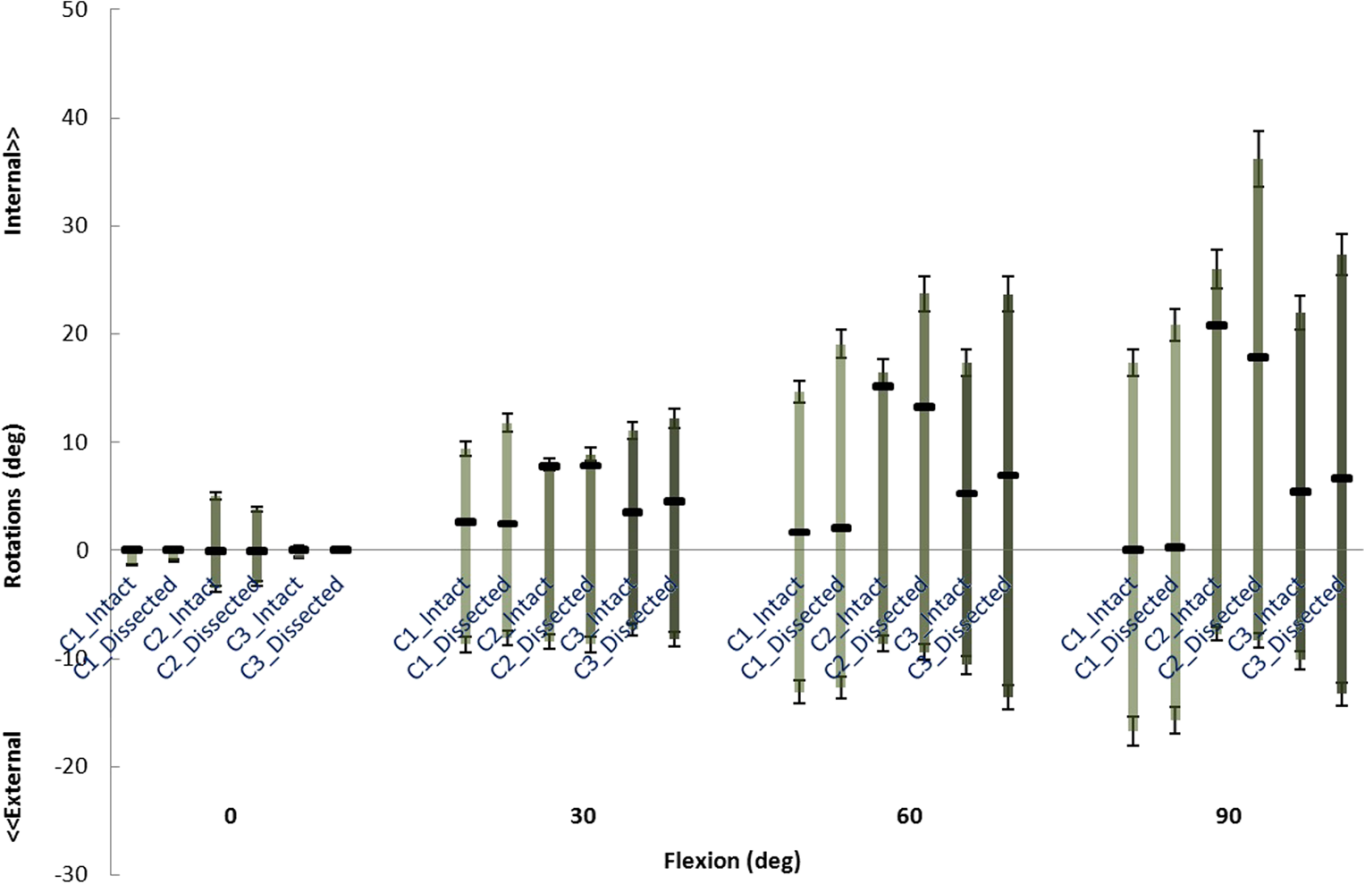
Internal-external rotations of three cadaveric knees at four flexion angles indicating unloaded joints (middle square), and with internal torque (upward bars) and external torque (downward bars)

Internal-external rotations of the first and third specimens were negligibly influenced by dissection during flexion, whereas the second specimen maximally showed an external rotational perturbation of 2.9° at 90° (Fig. 5).

Internal rotation increased by less than 1.2° after peripheral soft tissue removal for all specimens at full extension and 30° of flexion, except for the first knee (2.6° increase at 30°). At larger flexion angles, the laxity increased up to 12.9°.

Upon application of external rotation, the rotation of the first and second knees increased maximally by 1.7° after dissection, where in the third specimen, its rotations increased up to 5.7° at 60° flexion and 4.4° at 90° flexion.

#### Valgus-varus laxity

**Fig. 6:**
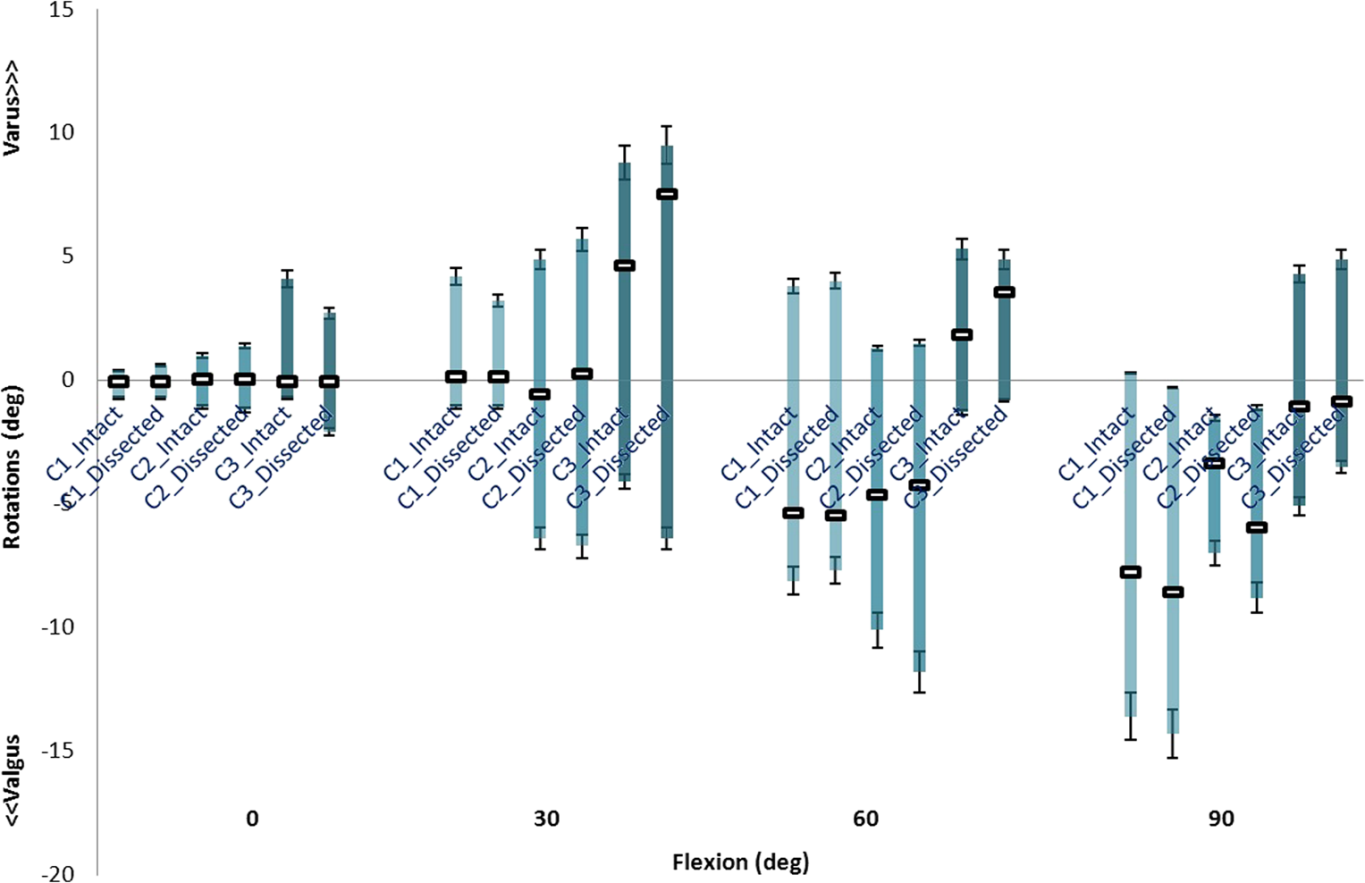
Varus-valgus rotations of three cadaveric knees at four flexion angles indicating unloaded joints (middle square), and with varus moment (upward bars) and valgus moment (downward bars)

In unloaded flexion, the first specimen showed only a slight valgus rotational increase at 90° by about 1.0° (Fig. 6). The second and third knees were inclined to more varus rotation at 30° and 60° of flexion, by less than 1.0° for the second knee and 2.9° (30° flexion) and 1.7° (60° flexion) for the third specimen. In 90° of flexion, only the second knee was considerably influenced by soft tissue removal (5.0° valgus).

Upon applying a varus moment, the maximum increase in varus rotational laxity occurred at 90° of flexion for the second specimen (3.0°), where for the first and third knees, it was less than 1.0° in all flexion angles.

### Finite element models

**Fig. 7:**
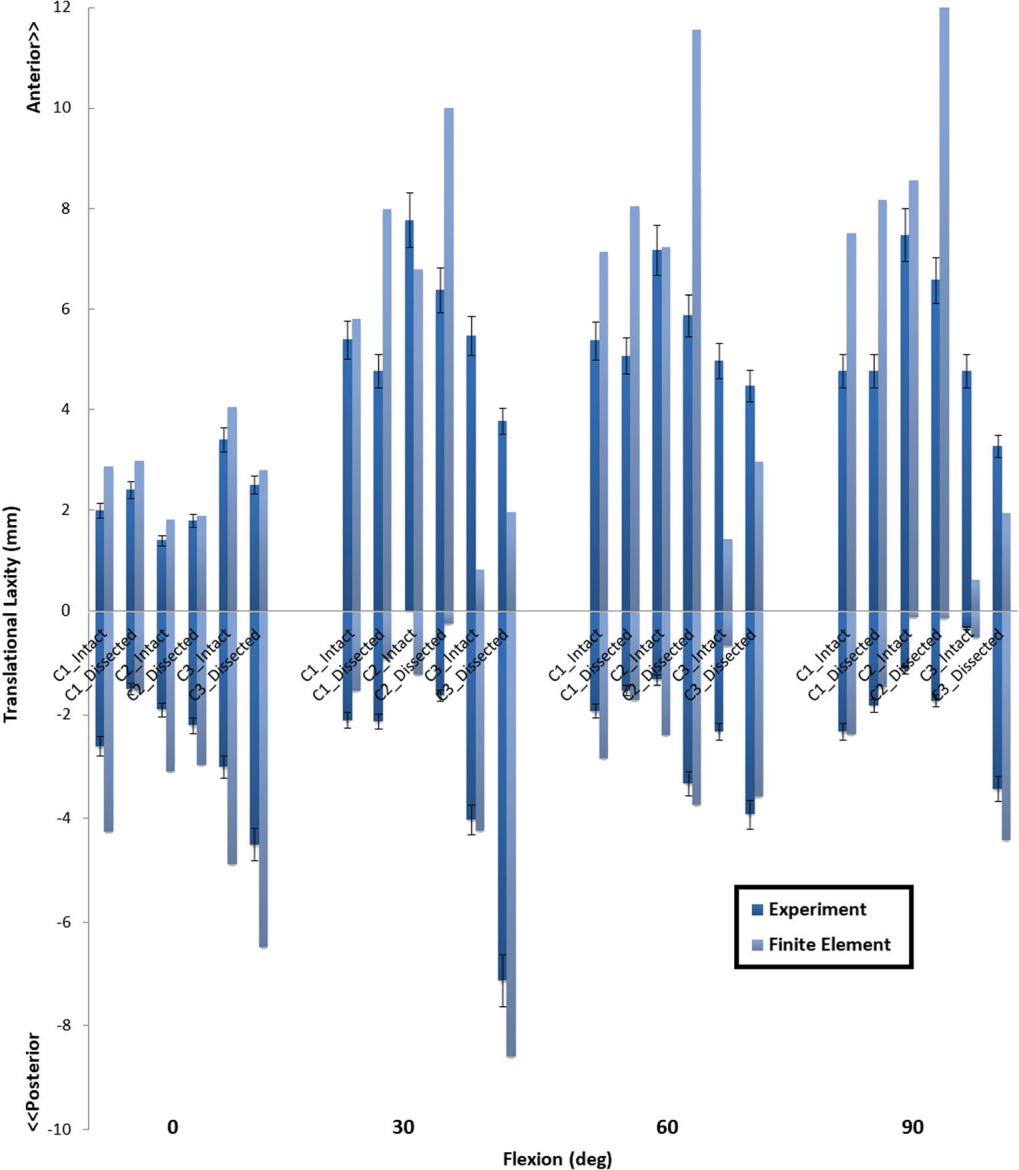
The anterior-posterior laxity predicted by FE models with (intact) and without (dissected) additional spring structures and measured in the experiment at different flexion angles

**Fig. 8:**
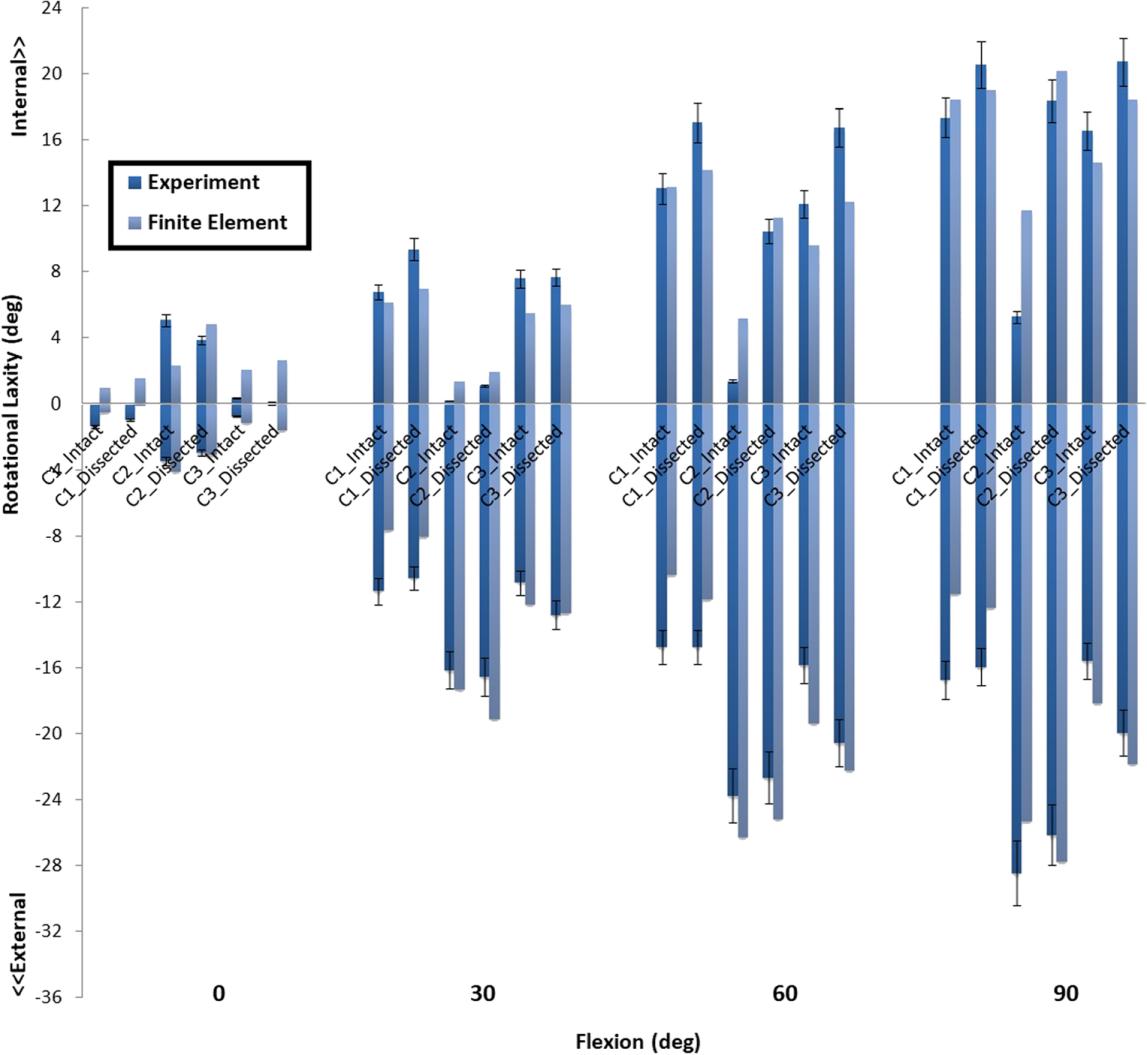
The internal-external rotational laxity predicted by FE models with (intact) and without (dissected) additional spring structures and measured in the experiment at different flexion angles

**Fig. 9:**
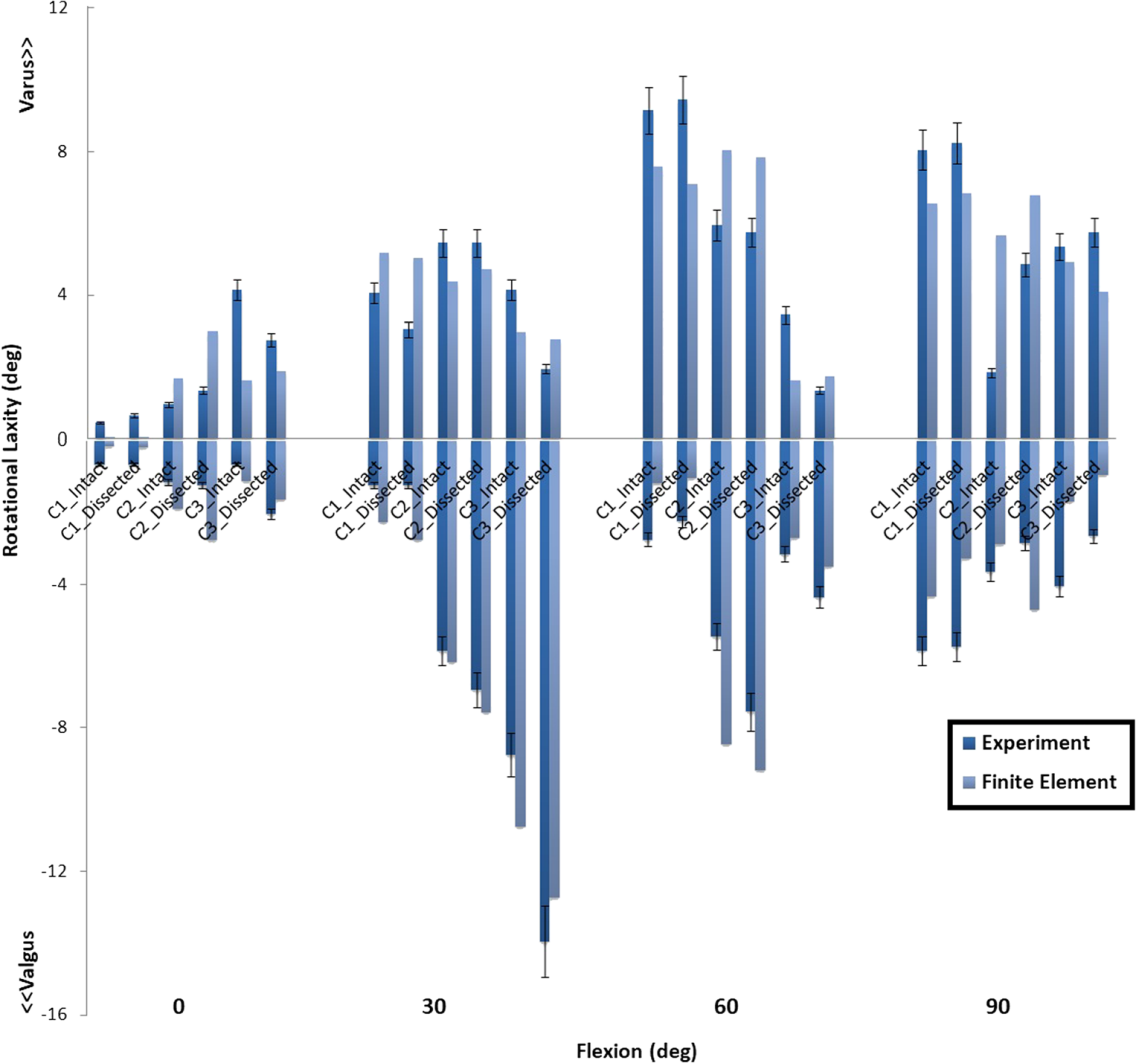
The valgus-varus rotational laxity predicted by FE models with (intact) and without (dissected) additional spring structures and measured in the experiment at different flexion angles

Table 2 shows the estimated stiffness for the modeled structures (APL, OPL, ALL, MCap, and LCap), with which the closest intact knee laxity was obtained for all three knee specimens. The laxity outcomes for FE models with and without the additional structures were compared with the experimental laxity results in Fig. 7 (anterior-posterior), Fig. 8 (internal-external), and Fig. 9 (valgus-varus).

**Table 2:** The spring stiffness of the five modeled structures to be representative for the dissected structures, in three subject-specific FE models

	The stiffness of the representative spring elements (N/mm)				
	APL	OPL	ALL	MCap	LCap
Initial value ± range	28 ± 14	28 ± 14	42 ± 26	15 ± 10	15 ± 10
C1	34	25	40	15	14
C2	40	30	45	19	17
C3	32	42	42	23	15

## Discussion

In the current study, the influence of the peripheral soft tissues and posterior capsule on knee joint laxity was investigated based on laxity tests in three human cadaveric specimens. Six different loading regimes were applied to each specimen pre- and post-dissection, at four different flexion angles. Based on the laxity outcomes, additional structures were modeled in three validated specimen-specific FE models to achieve the pre-dissection knee laxities.

Removal of the peripheral soft tissues only had a limited effect on the anterior-posterior laxity, but it did effect the neutral (unloaded) position of the joint. At larger flexion angles, the peripheral tissue provided substantial internal rotational constraints, but it did not change the neutral rotational position in an unloaded state. In lax knees, the peripheral tissues showed a limited influence on neutral valgus-varus rotations and valgus rotational laxity.

The implication of omission of the peripheral and posterior capsular tissue in knee models can therefore vary depending on the simulated task and the loading conditions. Werner et al. showed that contact distribution and contact loads on medial and tibial compartments significantly changed with a valgus-varus variation as little as 3° in gait, based on the experiment on four cadaveric implanted knees [33]. Similar findings of Engin et al. on human native knee joint confirm the high sensitivity of knee contact biomechanics to valgus-varus rotational configurations [34].

Our results indicate a valgus-varus change beyond 3° at flexion angles of 60° and 90° with peripheral tissues and posterior capsule removal. The change in internal-external laxity by ignoring the peripheral tissues can alter not only tibiofemoral joint behavior but also the biomechanics of patellofemoral joint. However, patellar kinematics and patellofemoral contact pressure were shown to be slightly more sensitive to internal rotation, where an internal rotational change of 5° can alter the patellofemoral joint biomechanical behavior [35]. The alteration in the posteriorly directed joint behavior by ignoring peripheral and capsular tissues can also lead to different cruciate ligament forces [5, 36]. According to Yao et al., an anterior-posterior perturbation of even 0.1 mm, which is less than what was measured in the current experiment, can lead to a considerable difference in tibiofemoral contact variables [37].

According to the study of Torzilli et al., the small difference between the intact and dissected knees at varus and external rotational and posterior translational mechanical loads could be attributed to the popliteofibular ligament [10]. They also reported a limited static mechanical resistance of the popliteal tendon in varus, more particularly at 30°, where the maximum varus difference occurred in the current study. In the study of Griffith et al., with loading conditions similar to the loads applied in the current study, a reduced internal and valgus rotational stiffness at low flexion was reported, in the knees with the OPL dissected [15].

In the subject-specific FE models of the three cadaveric knees used in this study, modeling only the main structures of the knee joint could not acceptably predict the pre-dissected knee laxity in the experiment. Adding APL, OPL, ALL, MCap, and LCap as spring elements with adjusted stiffness in FE models, however, improved the replication of the pre-dissected knee behavior.

The main limitation of this study was the low number of specimens, which makes it impossible to draw general conclusions from the results, except demonstration of the inter-specimen variation in the effect of peripheral soft tissue on joint kinematics. A second limitation is the fact that the current in vitro experiments were performed statically, while the in vivo dynamics may be different specifically, as it has been proposed that the popliteal tendon mostly acts dynamically to stabilize the knee joint [38]. A larger tensile force could be more representative for the physiological patellar muscle force and might influence the stability of the joint. However, it was previously shown that proportional larger quadriceps force would result in similar patellofemoral laxity patterns as the quadriceps loads applied in the current study [39]. In the FE models, the stiffness of the additional structures were manually adjusted, where following a more robust optimization routine could improve the stiffness estimation further. Nonetheless, even with the manual adjustment, the FE models revealed an improvement in the laxity prediction of pre-dissected knees.

## Conclusions

Our findings indicated that in lax knees, ignoring the posterior capsule and peripheral soft tissues in computational models of the knee joint may lead to higher anterior translations and limited alterations in valgus rotations at 90° during unloaded flexion. Excluding these structures from the models may also result in an increase in posterior translational and valgus and internal rotational laxities when the knee is flexed. Consequently, if the simulation contains any flexion under posterior, internal, and valgus loads or unloaded deep flexion, it is strongly recommended to incorporate the posterior capsule and peripheral tissue representations, as for instance incorporated in this study.
